# The Role of Turtles as Coral Reef Macroherbivores

**DOI:** 10.1371/journal.pone.0039979

**Published:** 2012-06-29

**Authors:** Christopher H. R. Goatley, Andrew S. Hoey, David R. Bellwood

**Affiliations:** 1 Australian Research Council Centre of Excellence for Coral Reef Studies, and School of Marine and Tropical Biology, James Cook University, Townsville, Queensland, Australia; 2 Red Sea Research Center, King Abdullah University of Science and Technology, Thuwal, Kingdom of Saudi Arabia; Leibniz Center for Tropical Marine Ecology, Germany

## Abstract

Herbivory is widely accepted as a vital function on coral reefs. To date, the majority of studies examining herbivory in coral reef environments have focused on the roles of fishes and/or urchins, with relatively few studies considering the potential role of macroherbivores in reef processes. Here, we introduce evidence that highlights the potential role of marine turtles as herbivores on coral reefs. While conducting experimental habitat manipulations to assess the roles of herbivorous reef fishes we observed green turtles (*Chelonia mydas*) and hawksbill turtles (*Eretmochelys imbricata*) showing responses that were remarkably similar to those of herbivorous fishes. Reducing the sediment load of the epilithic algal matrix on a coral reef resulted in a forty-fold increase in grazing by green turtles. Hawksbill turtles were also observed to browse transplanted thalli of the macroalga *Sargassum swartzii* in a coral reef environment. These responses not only show strong parallels to herbivorous reef fishes, but also highlight that marine turtles actively, and intentionally, remove algae from coral reefs. When considering the size and potential historical abundance of marine turtles we suggest that these potentially valuable herbivores may have been lost from many coral reefs before their true importance was understood.

## Introduction

Herbivory is widely recognised as a vital process for the health and resilience of coral reefs [1]–[3], mediating the competition for benthic space between algae and reef-building corals. When present in sufficient densities herbivores can maintain algal communities in a cropped state, preventing the proliferation and expansion of macroalgal communities [4], [5]. However, reductions in herbivory through both small-scale experimental exclusions and regional-scale overfishing have demonstrated that, when released from top-down control, algal assemblages can shift from highly productive algal turfs to less productive, late successional stage macroalgae such as *Sargassum* [1], [6]–[8]. As such, herbivores are widely viewed as a key component of the resilience of coral reefs [9], [10]. To date, quantitative studies of herbivory on coral reefs have focussed on the roles of fishes and/or urchins [4], [11], [12]. Few studies have considered the role of macroherbivores in coral reef ecosystem processes [9], [13].

Worldwide, almost all populations of marine macroherbivores have suffered drastic declines. For example, many marine turtle populations have been estimated to be below 10% of their historical baselines [14], [15], while estimates of sirenian (dugong and manatee) populations suggest they are less than 3.1% of baselines [15], [16]. By underestimating the historic anthropogenic impact on populations of sirenians and turtles, both the natural population densities and ecological roles of these species have, until quite recently, been largely overlooked [14]–[16]. At ‘baseline’ populations, many species of sea turtles may have had important ecological roles structuring their various prey communities [17]. Hawksbill turtles *Eretmochelys imbricata*, have been shown to structure sessile invertebrate communities [18]–[20], while green turtles, *Chelonia mydas*, appear to have been the primary herbivores of seagrass beds in the Caribbean [21] and may still structure seagrass communities when they are abundant [22].

Both green and hawksbill turtles have circumtropical distributions and occupy a range of habitats, including seagrass beds, coral and rocky reefs, and oceanic waters. Green turtles, while often considered to be consumers of seagrass [23], can contain substantial proportions of macroalgae characteristic of reef environments in their stomachs [24]–[27]. Reports of herbivory by adult hawksbill turtles are less common. Hawksbill turtles are primarily predators of sponges [18], [28] or other sessile invertebrates [19], [29], but may also consume small amounts of algal material in some areas [30], [31]. In all cases it is not certain if the algae are directly targeted or if they are removed from the benthos or floating algal rafts. Hawksbill and green turtles frequent coral reefs throughout their range but their role as herbivores in these ecosystems remains poorly understood [9], [20].

Within coral reef systems herbivores may be broadly categorised into two functional groups (i.e., grazers and browsers) based on the algal material they remove and consequently the roles they perform in reef processes [32], [33]. Grazers typically feed on algal turfs or epilithic algal matrix (EAM; [34]) and play an important role in helping reefs to resist shifts to alternate states and reassemble following disturbances [9], [10]. In contrast, browsing taxa feed on leathery macroalgae and play a potentially critical role in the reversal of shifts to macroalgal-dominance on coral reefs [33], [35]. On reefs, herbivorous species rarely fulfil both functional roles and there is often little redundancy within functional groups [9], [36]. Furthermore, recent studies have shown that many of these fishes have relatively small home ranges (<10ha; [37]–[40]), suggesting their functional impact may be spatially restricted. The morphological and taxonomic distinctness of turtles might suggest that they could play unique roles on reefs. Using observations taken during experimental habitat manipulations and transplanted macroalgal assays we present evidence to highlight the potential roles of marine turtles as both algal turf grazers and macroalgal browsers in coral reef environments.

## Methods

All procedures in this study were conducted according to the animal ethics guidelines of James Cook University, Townsville, (animal ethics approval number: A1522), and permitting requirements of the Great Barrier Reef Marine Parks Authority (permit number: G10/33755.1).

**Figure 1:**
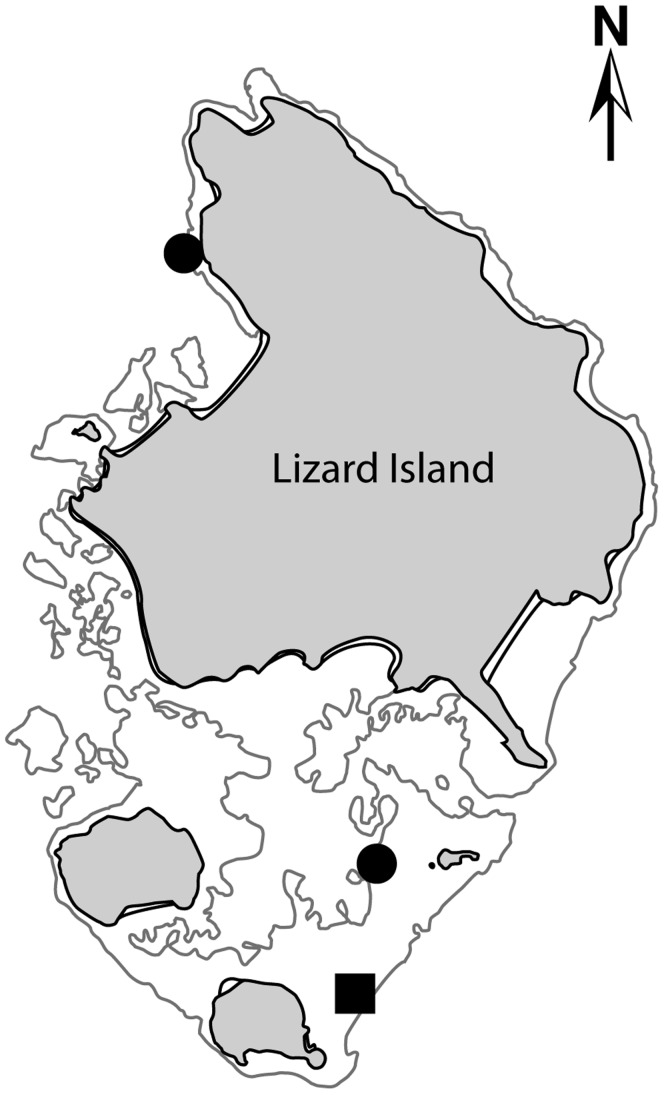
Site map The filled square represents the location of the sediment removal study, where green turtles (*Chelonia mydas*) were observed feeding. The filled circle to the north represents the sheltered reef base and to the south the back reef. At both sites hawksbill turtles (*Eretmochelys imbricata*) were observed feeding on transplanted *Sargassum* assays.

This study was conducted on the reefs surrounding Lizard Island (14°40′S 145°28′E), in the northern Great Barrier Reef (GBR; Fig 1). Two experimental manipulations: sediment reductions of algal turfs and macroalgal assays were performed to assess the ecological roles of coral reef grazers and browsers, respectively. While these experiments were primarily focused on the roles of herbivorous fishes [36], responses by marine turtles were also recorded and are reported herein. The sediment reductions were conducted on the exposed reef flat to the south-east of the island at a depth of 2–4 m, whilst the macroalgal transplants were conducted within six habitats of varying depth and wave exposure: the exposed reef crest (2–4 m depth), exposed reef flat (1–2 m), back reef (2–4 m), patch reef (4–6 m depth), sheltered reef flat (1–2 m) and sheltered reef base (6–8 m; Fig 1). See [36] for detailed description of habitats.

### Sediment Reduction

The exposed reef flat was selected as it has a high cover of EAM [12], [41] and is adjacent to the area of highest herbivory (i.e. exposed reef crest). The high sediment load of the EAM in this habitat has been proposed to limit grazing by reef fishes [42]. Two adjacent 0.75×1.5 m plots that were devoid of living coral and covered by EAM were temporarily delineated using a PVC frame. One of the plots was randomly selected and cleared of sediment using a compressed air powered airlift (hereafter ‘cleared plot’) while the adjacent plot was left undisturbed (hereafter ‘control plot’). Underwater video cameras (Sony DCR-SR100 HDD cameras in Ikelite housings), mounted on tripods, were then deployed for three hours to record the feeding activity of herbivores on the cleared and control plots simultaneously. Filming was continuous for the 3-hour experimental period with the PVC frame and a small scale bar being placed on the focal plane of the EAM in the experimental plots for approximately 10 s allowing calibration of experimental plots and turtle sizes from the video footage. This procedure was repeated until ten replicate plots had been recorded. Replicate plots were separated by at least 10 m.

The lengths of algal turfs within the plots were measured before and after the 3-hour experimental period. The heights of 20 randomly selected algal filaments were measured from both the cleared and control plots using the depth probe of vernier callipers. Data were analysed using a two-way fixed factor analysis of variance (ANOVA) followed by residual analysis to ensure assumptions of the test were met.

### Macroalgal Assays

To quantify browsing intensity across the six habitats a series of macroalgal assays were conducted. *Sargassum swartzii* (Ochrophyta: Phaeophyceae) was collected from an inshore reef in the Turtle Island Group (28 km west of Lizard Island). Similarly sized thalli (mean weight [± S.E.]  = 363.6±4.7 g) were transplanted to each of two haphazardly selected sites within each of the six habitats for 8-hours (approx. 07∶30–15∶30). Adjacent sites within each habitat were separated by a minimum of 50 m. Underwater video cameras (Sony DCR-SR100 HDD camera in Ikelite housing) mounted on concrete blocks were used to record feeding activity on the transplanted *Sargassum* for the 8- h experimental period within each habitat (see [36] for detailed description).

**Figure 2:**
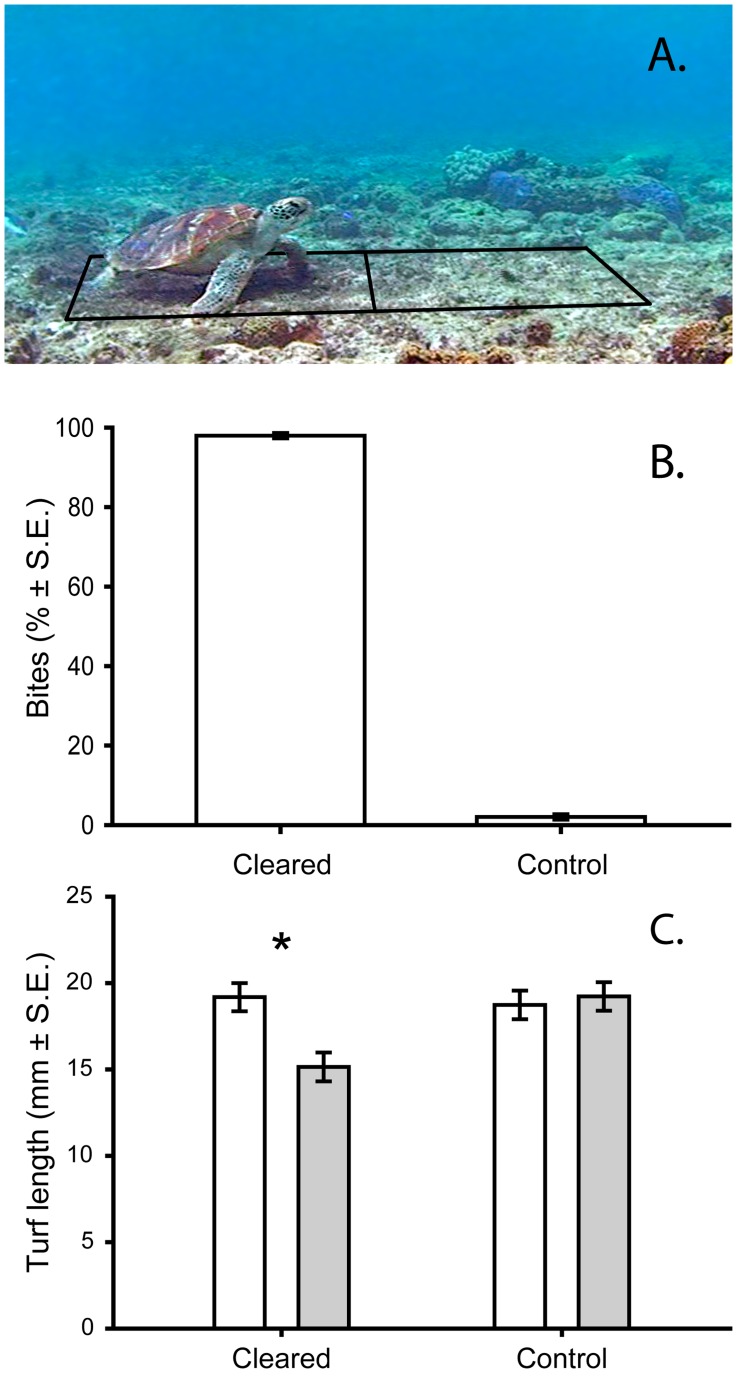
Grazing by the green turtle (*Chelonia mydas*) on sediment reduced plots. **A** Image capture of a green turtle feeding on the experimental sediment clearance plot. The lines represent the boundaries of the two adjacent plots: the sediment clearance plot to the left, and the control plot to the right. **B** The proportion of bites made in the cleared and control plots by green turtles during the three-hour experimental period (n  = 3 plots). **C** The reduction in turf length observed in three hours in the cleared and control plots respectively. White bars represent the mean initial length and grey the mean final length (n  = 3 plots for each treatment), * indicates a significant difference (Tukey’s HSD).

### Video Analyses

The video footage from both experimental manipulations was examined and all bites by turtles on the EAM within the cleared and control plots, and the transplanted *S. swartzii* thalli, were recorded. Turtles were identified to species and size (straight carapace length, SCL) was estimated. Throughout this study, results are presented as means ± standard error.

## Results

Reducing the sediment loads in the EAM on the exposed reef flat resulted in over forty times more grazing pressure on algal turfs by the green turtle, *Chelonia mydas* (Fig. 2a). In total 585 bites were recorded over nine feeding bouts on three of the ten experimental plots. Each feeding bout lasted approximately four and a half minutes (4∶36±0∶42; see video S1); between bouts the turtles were observed to remain near the plots but did not feed. Where turtles fed, grazing was overwhelmingly on the cleared plots (98.0±0.7% of bites; Fig. 2b). Size estimates of the green turtles observed feeding revealed at least three turtles were feeding on the plots, with a mean length of 56.0±4.5 cm (SCL).

**Figure 3:**
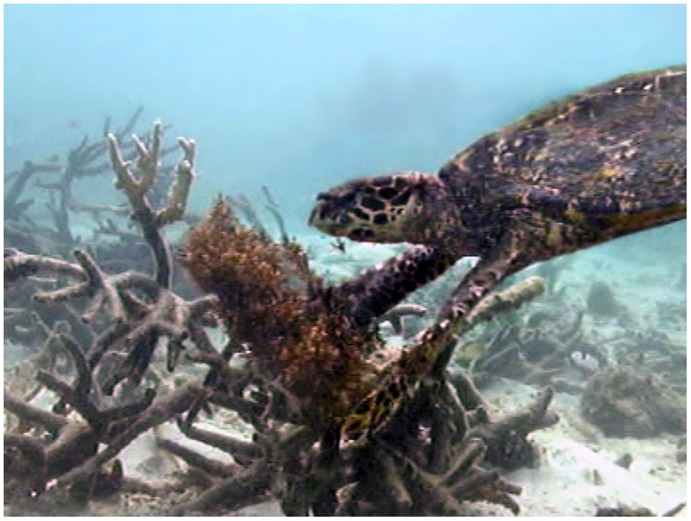
A hawksbill turtle (*Eretmochelys imbricata*) feeding on a transplanted thallus of the brown macroalga *Sargassum swartzii*.

The higher herbivory resulted in a 21.2% reduction in algal turf height in the three cleared plots grazed by turtles in just three hours (Fig. 2c; *F*_1, 232_ = 6.47, *p* = 0.01). Grazing by both herbivorous fishes and green turtles will have contributed to this reduction and it is impossible to attribute the cause of the reduction to either group, in fact, the reduction observed did not differ from that seen in plots without turtles (21.4%). Herbivorous reef fish, however, appeared to be deterred by the presence of turtles, and as such, the presence of a single turtle appeared to have comparable effects to multiple reef fishes. The large size and large number of bites made by turtles might indicate a possibly important role.

Analysis of 288 hours of video footage of the *S. swartzii* assays across the six habitats revealed that while most browsing was by fishes, two turtles were recorded feeding on the *Sargassum*. Both were hawksbill turtles, *E. imbricata*, (approx. 58 cm SCL), with one being recorded to feed on *Sargassum* in the back reef habitat, and the other in the sheltered reef base (Figs. 1, 3; videos S2 and S3). On each occasion the turtles took three bites from the *Sargassum*.

## Discussion

Our results provide observational evidence that marine turtles may function as both grazing and browsing herbivores on coral reefs. Collectively, green and hawksbills turtles displayed responses to sediment reductions of algal turfs and macroalgal assays that were remarkably similar to those of herbivorous fishes. While we were unable to isolate the effects of turtles from those of fishes in reducing the algal biomass, our observations clearly show that marine turtles are actively targeting and removing both algal turfs and leathery macroalgae from coral reef habitats. We postulate that, in doing so, turtles may potentially play a role in important ecological processes on coral reefs.

Numerous studies have advocated the importance of herbivory to reef health and resilience, focusing on the roles performed by herbivorous fishes [2]–[4], [9], [12], [36]. Fishes, while undoubtedly playing a key role in reef processes, lack some important morphological and behavioural traits of larger organisms. Larger animals, having larger mouths, usually take larger, more forceful, bites, and as such may be expected to have greater functional impacts [43], [44]. While the general scarcity of macroherbivores reduces the net benefits of size, larger individuals can offer greater ecological benefits in terms of mobility and may complement the roles of herbivorous fishes and urchins on coral reefs.

Ecological resilience may derive from overlapping function within scales, and reinforcement across scales [45]. Within coral reef systems, even some of the largest herbivorous reef fishes have relatively small home ranges. For example, the grazing steephead parrotfish, *Chlorurus microrhinos* (maximum length 80 cm) has a home range of less than 1 ha [40] and large browsing herbivores, such as the bluespine unicornfish, *Naso unicornis* (maximum length 70 cm) maintain home ranges well under 10 ha [37], [39]. As such, the ability of herbivorous reef fishes to respond to phase shifts appears somewhat limited by their spatial distribution; truly wide-ranging or roving reef herbivores may be rarer than previously assumed. Marine turtles, however, have far larger home ranges (over 3900 ha [46]) and often undergo large migrations [47]–[49]. We suggest that, regardless of phylogeny, large mobile herbivores with large home ranges could play any ecological roles on reefs at a broad scale, providing a function not yet observed on reefs.

### Grazing by Green Turtles, *Chelonia Mydas* on Coral Reefs

Green turtles made over forty times more bites on plots of algal turfs (EAMs) that had been subject to artificial sediment reduction than those with natural sediment loads. The observed behaviour of green turtles mirrors that seen by fishes in the only other study using sediment reductions on coral reefs [42]. Why sediment suppresses herbivory is unclear, but is likely to be associated with diminished accessibility of resources and reduced energy uptake per bite [50], [51]. Regardless of the cause, it appears that natural sediment loads in coral reef EAMs are enough to suppress herbivory on coral reefs across multiple herbivorous taxa.

The large size of green turtles compared to herbivorous reef fishes suggests they could play a greater functional role per-individual than herbivorous fishes. However, as turtles were not observed to respond differently to fishes, well-grazed reefs may not gain any potential benefits from increased turtle grazing. Larger turtle populations might not act as an alternative to current management techniques, only to supplement them.

Green turtles are well known as herbivores (Table S1), however most studies have used gut contents data to determine what algae the turtles have consumed. While analysis of gut contents clearly indicates what the study animal has ingested, it provides no indication of the source of the prey. As such, the propensity of green turtles to feed on coral reefs has remained unclear. The source of ingested algae could have been non-reefal hard substrates or flotsam. Furthermore, grazing of algal turfs is likely to be under-reported in gut contents analyses, as algal filaments are small and difficult to identify. Our observations demonstrate that green turtles do actively consume turf algae from coral reef environments. Furthermore they respond to turf-associated sediment in a manner comparable to fishes [42].

### Browsing by Hawksbill Turtles, *Eretmochelys Imbricata* on Coral Reefs

Although few bites were videoed, the evidence presented herein supports previous observations by the authors of turtles feeding on leathery macroalgae on both algal dominated inshore reefs and offshore coral dominated reefs on the GBR. Previous studies using gastric lavages or dissections have demonstrated that sponges and other sessile invertebrates account for over 95% of the diet of *E. imbricata*, with macroalgae only representing a very minor component (Table S2). However, similarly to those for green turtles, these studies do not provide information on the source of the small proportions of macroalgae ingested by hawksbill turtles, which could have been ingested incidentally when feeding on sponges or from floating algal mats. Our observations indicate that adult hawksbill turtles may be actively targeting and ingesting leathery macroalgae when available on coral reefs.

### Historical Roles, Shifting Baselines and Generalist Herbivory

The concept of shifting baselines on reefs [52], [53] is of particular relevance when considering macroherbivores as they show some of the quickest declines after human exploitation [54]. Marine turtles, along with most marine macrofauna have been hunted for considerably longer than their populations have been monitored; as such, estimates of historical populations are hard to derive and highly variable [14], [15], [55].

Regardless of the actual figures, turtle populations have suffered considerable depletion worldwide [56]–[58]. Only after the depletion of Caribbean green turtle populations did their role as the principal grazer of seagrass communities become apparent (cf. [23]). Some modern populations of turtles can control and even overgraze some marine habitats [22], further highlighting that even at low densities turtles have the potential to play significant roles in marine ecosystems.

We speculate that, although each species was only seen to feed on one functional group of algae (green turtles on EAMs and hawksbills on leathery macroalgae), and in combination with previous reports of the diets of marine turtles (Tables S1 and S2), contrary to most fish taxa, marine turtles might represent a trophically flexible group of herbivores on coral reefs. As the resilience of coral reefs is often reliant on numerous roles provided by individual species or small functional groups [10], [36], [59], large generalist herbivores would be particularly valuable, as they would provide a measure of redundancy across a wide array of functions. Reported dietary shifts with changes in prey abundances by turtles highlight their potential as ecological stand-ins [60]. At current population densities, however, the scarcity of turtles means that, even if turtles were generalist herbivores they would be unlikely to be able to replace any lost ecosystem function. Furthermore, the capacity for fishes to take over the roles of turtles is limited [35]. Of the 60–70 species of nominally herbivorous fishes found on the Great Barrier Reef less than 10 have been found to feed on leathery macroalgae, and in each case they have highly restricted diets [35], [61]. Turtles may, therefore, provide a unique combination of size, mobility and dietary flexibility that is unlikely to be matched by fishes.

While recent conservation efforts have yielded encouraging results [57], threats to turtles are not restricted to hunting and bycatch. Changing environmental conditions and sea level rise are a particular threat to turtles, which are reliant on specific nesting beaches and have temperature dependent sex ratios [62]–[64]. These threats are far more challenging to manage than those from exploitation and the future for turtle populations remains uncertain. The population declines and continuing threats to marine turtles may, therefore, mean that coral reefs might have lost, what may be, their largest generalist macroherbivores before their role was fully understood.

## Supporting Information

Video S1 **Green turtle (** ***Chelonia mydas*** **) feeding on algal turfs.** The darker area to the left of the frame is cleared of sediment.
(MOV)

Video S2 **Hawksbill turtle (** ***Eretmochelys*** **imbricata), feeding on** ***Sargassum swartzii*** **assay at the back reef (see** **Fig. 1** **).**
(MOV)

Video S3 **Hawksbill turtle (** ***Eretmochelys*** **imbricata), feeding on** ***Sargassum swartzii*** **assay at the sheltered reef base (see** **Fig. 1** **).**
(MOV)

Table S1 **Summary of previous dietary studies of the green turtle,** ***Chelonia mydas*** **.** Values represent percent volume of each dietary category. Where quantitative estimates were not available †† indicates the dominant component, and * indicates presence as a minor component. Literature cited referenced in Appendix S1.
(PDF)

Table S2 **Summary of previous dietary studies of the hawksbill turtle,** ***Eretmochelys imbricata*** **.** Values represent percent volume of each dietary category. Where quantitative estimates were not available †† indicates the dominant component, and * indicates presence as a minor component. Literature cited referenced in Appendix S1.
(PDF)

Appendix S1 **References cited in tables S1 and S2.**
(DOC)
