# Supplementary material for: The Role of Turtles as Coral Reef Macroherbivores
Source: PLoS One. 2012 Jun 29;7(6):e39979. doi: 10.1371/journal.pone.0039979 (PMC3386948; doi:10.1371/journal.pone.0039979)
Supplement: Table S1 — Summary of previous dietary studies of the green turtle, Chelonia mydas . Values represent percent volume of each dietary category. Where quantitative estimates were not available †† indicates the dominant component, and * indicates presence as a minor component. Literature cited referenced in Appendix S1. (PDF) [file pone.0039979.s004.pdf]

Table S4. Summary of previous dietary studies of the green turtle, *Chelonia mydas*. Values represent percent volume of each dietary category. Where quantitative estimates were not available †† indicates the dominant component, and * indicates presence as a minor component. Literature cited referenced in S6.

					**Macroalgae**					
**Life stage**	**Method**	**Sample Size**	**Seagrass**	**Chlorophyta**	**Rhodophyta**	**Phaeophyta**	**Sponge**	**Other Invertebrate**	**Location**	**Source**
Juvenile	Lavage	113	59.7	9.3	26.3				Moreton Banks, Queensland	Read & Limpus 2002
Juvenile	Lavage	127	25.5	14.3	62.7	1.8			Flathead Gutter, Queensland	Read & Limpus 2002
Juvenile	Lavage	20	45.7		47.3			6.8	Moreton Bay, Queensland	Brand-Gardner et al. 1999
Juvenile	Lavage	40	0.1	4	89.9	0.7	0.4	0.8	Florida	Gilbert et al. 2008
Juvenile	Lavage	108	81.8	0.5	13.9	0.9		0.5	Green Island, GBR	Fuentes et al. 2006
Subadult	Lavage	15		49.4	49.9		0.6		Baja California, Mexico	Lopez-Mendilaharsu et al. 2008
Subadult	Lavage	191	2.1	11.6	78	1.7		0.9	Hawaii	Arthur & Balazs 2008
Subadult /Adult	Lavage	146	85.5	< 1.0	9.4	< 1.0	< 1.0	1.3	Shoalwater Bay, Qld	Arthur et al. 2009
Subadult /Adult	Stomach content	243	88.6	3.1	4.8	0.3	0.9	0.4	Nicaragua	Mortimer 1981
Subadult /Adult	Lavage	408		30	38.6	29.5			Heron Island, GBR	Forbes 1996
Subadult /Adult	Stomach content	9	> 50	ca 15		ca 10			Oman	Ross 1985
Subadult /Adult	Crop contents	372	ca 4	ca 20	ca 65	ca 7			Kane'ohe Bay, Hawaii	Russell & Balazs 2009
Subadult /Adult	Lavage & faecal	101		4.4	90			2.9	Gulf of California, Mexico	Seminoff et al. 2002
Subadult Adult	Lavage	65		43.3	33.1	5.8		8.8	Galapagos Islands	Carrion-Cortez et al 2010
Adult	Stomach content	1		††		*			Tokelau	Balazs 1983

Summary of
