# Supplementary material for: The Role of Turtles as Coral Reef Macroherbivores
Source: PLoS One. 2012 Jun 29;7(6):e39979. doi: 10.1371/journal.pone.0039979 (PMC3386948; doi:10.1371/journal.pone.0039979)
Supplement: Table S2 — Summary of previous dietary studies of the hawksbill turtle, Eretmochelys imbricata . Values represent percent volume of each dietary category. Where quantitative estimates were not available †† indicates the dominant component, and * indicates presence as a minor component. Literature cited referenced in Appendix S1. (PDF) [file pone.0039979.s005.pdf]

Table S5. Summary of previous dietary studies of the hawksbill turtle, *Eretmochelys imbricata*. Values represent percent volume of each dietary category. Where quantitative estimates were not available †† indicates the dominant component, and * indicates presence as a minor component. Literature cited referenced in S6.

					**Macroalgae**					
**Life stage**	**Method**	**Sample Size**	**Seagrass**	**Chlorophyta**	**Rhodophyta**	**Phaeophyta**	**Sponge**	**Other Invertebrate**	**Location**	**Source**
Juvenile	Photograph	NA					††	*	Cayman Islands	Blumenthal et al. 2009
Subadult /Adult	Stomach content	64					95.3		Caribbean	Meylan 1988
Subadult /Adult	Lavage	75					††	*	Puerto Rico	van Dam & Diez 1997
Subadult /Adult	Lavage	18		1.6	3.8	0.2	0.4	94	US Virgin Islands	Mayor et al 1998
Subadult /Adult	Lavage	146		1.2	2.1	0.7	91.6	4.8	Cuba	Alvarez 2000
Adult	Direct Observation	1						††	Southeastern Brazil	Stampar et al. 2007
Adult	Direct Observation	1						††	Aldabra Atoll	Obura et al 2010
