# Supplementary material for: The Role of Turtles as Coral Reef Macroherbivores
Source: PLoS One. 2012 Jun 29;7(6):e39979. doi: 10.1371/journal.pone.0039979 (PMC3386948; doi:10.1371/journal.pone.0039979)
Supplement: Appendix S1 — References cited in tables S1 and S2. (DOC) [file pone.0039979.s006.doc]

**LITERATURE CITED IN TABLE S4 & S5.**

Alvarez BLA (2000) Hawksbill turtle feeding habitats in Cuban waters. In: Abreu-Grobois FA, Briseño-Dueñas R, Márquez R, Sarti L (compilers) Proceedings of the Eighteenth International Sea Turtle Symposium. US Dep Commer NOAA Tech Memo NMFS-SEFSC 436: 65

Arthur KE, Balazs GH (2008) A Comparison of Immature Green Turtle (*Chelonia mydas*) Diets among Seven Sites in the Main Hawaiian Islands. Pac Sci 62: 205-217

Balazs G (1983) Sea turtles and their traditional usage in Tokelau. Atoll Res Bull 279: 1–29

Blumenthal JM, Austin TJ, Bell CDL, Bothwell JB, Broderick AC, et al. (2009) Ecology of Hawksbill Turtles, *Eretmochelys imbricata*, on a Western Caribbean Foraging Ground. Chelonian Conserv Biol 8: 1-10

Brand-Gardner SJ, Lanyon JM, Limpus CJ (1999) Diet selection by immature green turtles, *Chelonia mydas*, in subtropical Moreton Bay, south-east Queensland. Aust J Zoo 47: 181-191

Carrion-Cortez JA, Zarate P, Seminoff JA (2010) Feeding ecology of the green turtle (*Chelonia mydas*) in the Galapagos Islands. J Mar Biol Assoc UK 90: 1005-1013

Forbes GA (1996) The diet and feeding ecology of the green sea turtle (*Chelonia mydas*) in an algal-based coral reef community. Ph.D thesis, James Cook University, Townsville, pp. 117-141

Fuentes MMPB, Lawler IR, Gyuris E (2006) Dietary preferences of juvenile green turtles (*Chelonia mydas*) on a tropical reef flat. Wildlife Research 33: 671–678

Gilbert EI, Ehrhart LM, Valdes EV, Walters LJ (2008) Juvenile green turtle (*Chelonia mydas*) Foraging ecology: feeding selectivity and forage nutrient analysis.

NOAA Tech Mem NMFS-SEFSC 582: 89

Léon YM, Bjorndal KA (2002) Selective feeding in the hawksbill turtle, an important predator in coral reef ecosystems. Mar Ecol Prog Ser 245: 249-258

Lopez-Mendilaharsu M, Gardner SC, Riosmena-Rodriguez R, Seminoff JA (2008)

Diet Selection By Immature Green Turtles (*Chelonia mydas*) at Bahia Magdalena Foraging Ground in the Pacific Coast of the Baja California Peninsula, Mexico.

J Mar Biol Assoc UK 88: 641-647

Mayor PA, Phillips B, Hillis-Star Z-M (1998) Results of the stomach content analysis on the juvenile hawksbill turtles of Buck Island Reef National Monument, U.S.V.I. In: Epperly SP, Braun J (compilers) Proceedings of the seventeenth annual sea turtle symposium. NOAA Tech Memo NMFS-SEFSC 415: 244-247

Meylan A (1988) Spongivory in hawksbill turtles: a diet of glass. Science 239: 393-395

Mortimer JA (1981) The Feeding Ecology of the West Caribbean Green Turtle (*Chelonia mydas*) in Nicaragua. Biotropica 13: 49-58

Obura DO, Harvey A, Young T, Eltayeb MM, von Brandis R (2010) Hawksbill turtles as significant predators on hard coral. Coral Reefs 29: 759

Read MA, Limpus CJ (2002) The green turtle, *Chelonia mydas*, in Queensland: feeding ecology of immature turtles in Moreton Bay, Southeastern Queensland. Mem Queensl Mus 48: 207-214

Ross JP (1985) Biology of the green turtle, *Chelonia mydas*, on an Arabian feeding ground. J Herpetol 19: 459-468

Russell DJ, Balazs GH (2009) Dietary shifts by green turtles (*Chelonia mydas*) in the Kane‘ohe Bay region of the Hawaiian Islands: a 28 year study. Pac Sci 63: 181-192

Seminoff JA, Resendiz A, Nichols WJ (2002) Diet of East Pacific Green Turtles (*Chelonia mydas*) in the Central Gulf of California, México. J Herpetol 36: 447-453

Stampar SN, da Silva PF, Luiz Jr. OJ (2007) Predation on the zoanthid *Palythoa caribaeorum* (Anthozoa, Cnidaria) by a hawksbill turtle (*Eretmochelys imbricata*) in Southeastern Brazil. Mar Turtle Newslett 117: 3-5

van Dam RP, Diez CE (1997) Predation by hawksbill turtles on sponges at Mona Island, Puerto Rico. Proc 8th Int Coral Reef Sym 2: 1421-1426
